# Development, Feasibility, Acceptability, and Utility of an Expressive Speech-Enabled Digital Health Agent to Deliver Online, Brief Motivational Interviewing for Alcohol Misuse: Descriptive Study

**DOI:** 10.2196/25837

**Published:** 2021-09-29

**Authors:** Maya Boustani, Stephanie Lunn, Ubbo Visser, Christine Lisetti

**Affiliations:** 1 Department of Psychology Loma Linda University Loma Linda, CA United States; 2 Knight Foundation School of Computing and Information Sciences Florida International University Miami, FL United States; 3 Department of Computer Science University of Miami Miami, FL United States

**Keywords:** digital health agent, virtual health assistant, online intervention, alcohol abuse, brief intervention, motivational interviewing, intelligent virtual agent, embodied conversational agent

## Abstract

**Background:**

Digital health agents — embodied conversational agents designed specifically for health interventions — provide a promising alternative or supplement to behavioral health services by reducing barriers to access to care.

**Objective:**

Our goals were to (1) develop an expressive, speech-enabled digital health agent operating in a 3-dimensional virtual environment to deliver a brief behavioral health intervention over the internet to reduce alcohol use and to (2) understand its acceptability, feasibility, and utility with its end users.

**Methods:**

We developed an expressive, speech-enabled digital health agent with facial expressions and body gestures operating in a 3-dimensional virtual office and able to deliver a brief behavioral health intervention over the internet to reduce alcohol use. We then asked 51 alcohol users to report on the digital health agent acceptability, feasibility, and utility.

**Results:**

The developed digital health agent uses speech recognition and a model of empathetic verbal and nonverbal behaviors to engage the user, and its performance enabled it to successfully deliver a brief behavioral health intervention over the internet to reduce alcohol use. Descriptive statistics indicated that participants had overwhelmingly positive experiences with the digital health agent, including engagement with the technology, acceptance, perceived utility, and intent to use the technology. Illustrative qualitative quotes provided further insight about the potential reach and impact of digital health agents in behavioral health care.

**Conclusions:**

Web-delivered interventions delivered by expressive, speech-enabled digital health agents may provide an exciting complement or alternative to traditional one-on-one treatment. They may be especially helpful for hard-to-reach communities with behavioral workforce shortages.

## Introduction

### Background

Alcohol use disorder (AUD) affects 10%-20% of men and 5%-10% of women over their lifetime, and 26.4% of adults engage in binge drinking. AUD is the third leading preventable cause of death [1], with driving under the influence accounting for 31% of driving fatalities. In addition to personal costs associated with AUD, alcohol abuse costs the US economy an average of $249 billion per year. Motivational interviewing (MI) [2] is an effective and scalable intervention for AUD [3]. It is a client-centered counseling style that is directive and elicits behavior change by helping clients explore ambivalence and resolve it in order to develop *intrinsic* motivation to change. Adaptations of MI have bourgeoned to meet the need for motivational interventions that are brief and thus compatible within primary care settings [4]. Brief motivational interviewing (BMI) interventions include MI’s style of communication (communicating empathy, increasing discrepancy, rolling with resistance, and supporting self-efficacy) with the common underlying elements of effective brief interventions (eg, feedback, menus of options for changing respectful of current readiness to change, supportive advice). BMI can be delivered in multiple settings, as both a standalone intervention and in combination with other strategies for substance use disorders, such as cognitive-behavioral therapy, and has been found to be effective across meta-analyses [3-5]. Despite the high rates of alcohol use and availability of these effective interventions, only 1 in 10 individuals with AUD receive care [6,7].

### Barriers to Care

A number of barriers prevent individuals from accessing the treatment they need, including acknowledging the need for treatment [8], availability of trained providers [9], proximity of providers, access to transportation, affordability, insurance coverage, scheduling, and stigma [10]. Individuals living in rural settings or in poverty — where alcohol abuse is more prominent — are disproportionately impacted by these barriers [9]. In rural settings in particular, anonymity is more difficult [11] and increases stigma around help-seeking. Lack of flexible scheduling options for individuals who work full time further exacerbate barriers to treatment [9]. Finally, when individuals do access treatment, it is not always an evidence-based treatment — further complicating issues around access to quality care.

### Digital Health Interventions

Digital health interventions (DHIs) are interventions that are delivered via digital platforms (eg, applications, websites, mobile devices). Unlike telehealth (where a live provider meets with a consumer via a video chat), DHIs do not rely on a human provider to deliver services. As such, they have the potential to reduce a number of barriers associated with location (can be accessed from anywhere), scheduling (can be accessed at any time), stigma (can be accessed anonymously from the privacy of one’s home), and cost (most are affordable or free). Past research indicates that consumers tend to be more truthful when disclosing possibly stigmatizing information such as a drug or alcohol disorder to a computer versus a human [12-16] — providing another advantage to DHIs as they can be more informed about consumers’ at-risk behaviors than a human provider.

A review of DHIs [17] indicates that these interventions range from brief screening tools to several months of structured activities. Content includes screening and self-monitoring, personalized normative feedback, goal-setting activities, and interactive journaling. Benefits include reductions in alcohol consumption and consequences of heavy drinking. Despite all the benefits associated with DHIs, they are associated with high dropout rates [18]. For instance, a systematic review of the use of mental health support smartphone applications indicates that only 4% of users engage daily with the applications [19]. Researchers suggest that the use of a DHI without the support or recommendation of a mental health professional may limit its use [19,20]. Mohr et al [21] pointed out that improvement in mental health conditions tends to require continued behavior change over many weeks or months, yet mental health technologies are mainly didactic, thereby not ideal for supporting engagement and behavior change. Most behavioral health technologies require some human backing from a mental health professional to sustain engagement. Qualitative studies point to lack of motivation due to frustrating technology, inadequate content, competing priorities, and lack of face-to-face encounters [22,23]. This limits the promise of DHIs as a scalable solution to increase access to care, which our approach aims to improve.

A review of DHIs designed specifically for MI [24] further points out that, given the important emphasis on the interpersonal therapeutic communication style that is a core aspect of MI, delivering MI through these different modalities is difficult. For instance, can the “MI spirit,” or relational aspects, happen digitally? Therefore, comprehending the type of technology used to deliver relationally focused treatments provides an understanding of how technology may be used to replace face-to-face contact. The study found that DHIs for MI vary greatly in terms of technology and richness of the media used, ranging from text-only to audio files, video files, and interactive animated characters, and that the most common feature of these technologies was personalized feedback to the participant based on their input. Only a subset of a few programs used videos (eg, a “video-doctor” actor playing a doctor’s responses in MI style) or animations (eg, a robot’s head with facial expressions supporting participants during the intervention). These media were always developed with the aim to mirror interpersonal communication. Our current focus on developing and evaluating 3D virtual characters able to deliver a BMI, with appropriate facial expressions, body gestures, speech synthesis, and speech recognition in real time, aims at providing awareness into how technology may be used to replace face-to-face contact.

The review by Shingleton and Palfai [24] also found that, while surveyed articles explained methods for some aspects (eg, automatic computer prompts, chat rooms, emails, videos, animated characters) to deliver MI, most articles did not explain how they translated MI principles into the DHI nor whether or how the relational components were resolved. Translating aspects that require the MI spirit such as “expressing empathy” or “collaboration” to technology — versus consolidating commitment to change and developing discrepancy, on which most studies focused — is particularly difficult to implement in a piece of software. One conclusion was that future researchers need to detail both, not only how the technical aspects (eg, chat rooms, emails) are delivered but also how the relational aspects (eg, emoticons, videos of talking narrators) are delivered in order to increase the human-like discourse with the DHI. Asking questions to help understand how participants felt about and during the interaction were also encouraged as important “soft” outcomes to uncover ways to increase the “spirit” of MI within technology. As highlighted by Mohr et al [21], while usability testing has increased in recent years, the design of DHIs has generally not included input from end users. Our focus on technology outcomes in this article aimed at providing insight into these “soft” outcomes, by explicitly asking users of our DHI-specific survey questions about their experience with an expressive, animated embodied conversational agent (ECA) in order to inform the impact of our DHI current design, our future redesigns, and other researchers’ DHIs.

### Embodied Conversational Agents

ECAs (also known as virtual intelligent agents or virtual humans) are simulated human characters that may have the potential to increase consumer engagement in DHIs [25]. Unlike avatars — which are virtual entities that represent and are controlled by the user (popular in video gaming) — ECAs are virtual entities of their own that interact with a consumer autonomously and anonymously. They are developed with the aim to look, sound, and behave as closely to humans as possible. Their ability to hold conversations is still limited [26] but advancements in natural language processing and artificial intelligence (AI) hold promise in the future of ECAs as an alternative solution to traditional therapy for mental health and substance abuse concerns [27]. ECAs have the ability to use sophisticated multimodal communication to build rapport [28-41], communicate empathically [32-35,38], and engage in social talk [42-46]. Despite the promise of ECAs, research around the acceptability, feasibility, and utility of such technology by consumers of behavioral health interventions is limited. Exceptions can be found in a few studies using 3D ECAs with realistic animated facial expressions and mirroring of the users’ facial expressions [25], a study including an ECA with a dialog management system allowing users to answer freely to the ECA (albeit without full robustness for broad dissemination without synchronous human support) [26], and a few others using simple ECAs [43,47-50].

In spite of their success, however, ECA development did not scale with the now abundant internet devices (smartphones, laptops) and the latest progress in 3D graphics. Some attempts have been made to build web-based, 3D ECAs [51-53]. However, their implementation is still very basic, and they do not offer an integrated framework for web-based ECA development, including the ability to recognize and synthesize social cues in real time during spoken dialog, which is a significant technical challenge and which our ECA provides.

### Current Study

This study aimed to fill the gap in knowledge of using ECAs in behavioral health contexts by establishing the acceptability, feasibility, and utility of using ECAs by consumers undergoing a BMI intervention for alcohol abuse. BMIs are highly structured (*assessment* of, followed by normative *feedback*, then *menu* of change options), making them amenable to delivery via DHI [22], particularly if the “MI spirit,” or relational components, can be captured without face-to-face contact. One such BMI, namely the Drinker’s Check-Up (DCU) [2] is the intervention used for this work. DCU has been computerized as a menu-based, text-only program delivered online that targets alcohol abuse, reducing drinking by an average of 50% at a 12-month follow-up [50]. The DCU is one of the 2 English-language, web-based DHIs designed for the public that have been tested in randomized controlled trials (RCTs) [7]. We therefore chose to study how the delivery of the DCU by an ECA will be perceived by its users, given that its nonverbal and other media features aim to address the observed limitations of the use of avatars in DHIs for MI that do not strengthen the social relationship with the user [50].

**Figure 1:**
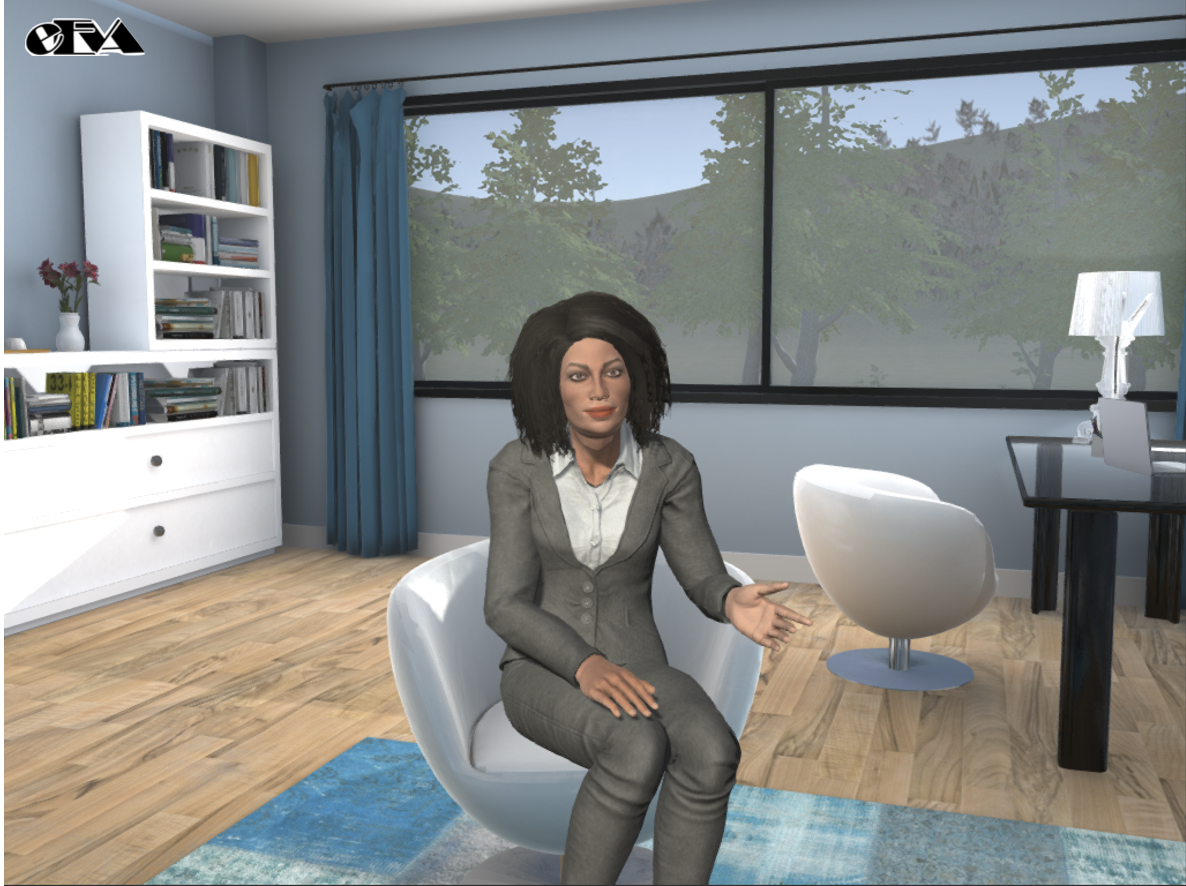
Our empathic embodied virtual agent (eEVA) delivering a brief motivational interviewing behavior change session.

**Figure 2:**
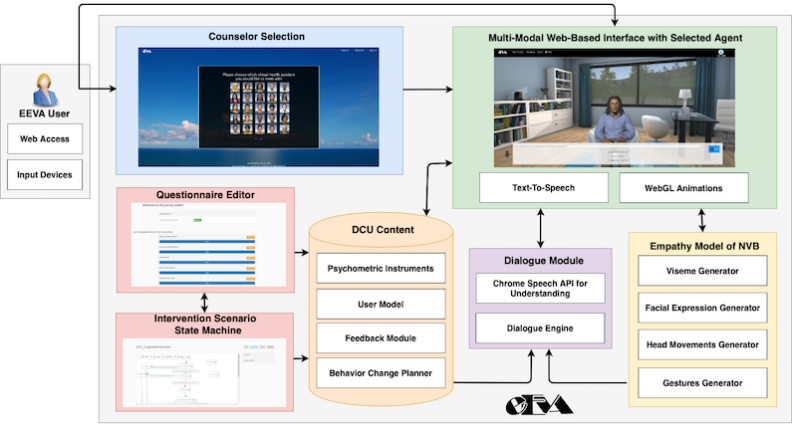
Empathic embodied virtual agent (eEVA) system overview. API: application programming interface; DCU: Drinker’s Check-Up; NVB: nonverbal behavior.

Using the technology acceptance model (TAM) [54,55] to guide our work, this study enabled us to determine if the ECA designed by our team using the empathic embodied virtual agent (eEVA; see Figure 1 and Figure 2) framework for building digital health agents [56] has enough personal characteristics and social abilities (eg, open-minded, supportive, respectful, friendly) to give users a positive experience (acceptability). The TAM stipulates that user acceptance can be predicted by the perceived usefulness (utility) and perceived ease of use (feasibility) of the technology. As such, we were interested in learning more about consumers’ perceived positive experience (acceptability), usefulness of eEVA (utility), and ease of use (feasibility) to better understand acceptability and potential for adoption of the technology. Having technology that consumers like and find easy to use and helpful increases the potential for adoption, which, in turn, increases access to care. Increases in access to care have the potential to improve health outcomes for alcohol users. Prior studies have found that MI for alcohol use (including online delivery via textual interface) improves health outcomes [57].

## Methods

### Intervention

#### DCU

The intervention is based on DCU — an evidence-based intervention that uses strategies from MI [57]. First, users provide detailed information about their drinking (eg, own drinking patterns or issues, family history of alcohol use). Next, they receive individualized feedback about their drinking habits, including information about risk factors and consequences [58]. Finally, they resolve their ambivalence about whether to change their drinking, plan for a change, and set goals for change. The intervention provides resources to help users with changing their drinking [59]. However, the DCU does not tell the clients what to do or not to do — it is up to the user whether they want to change their alcohol use. The DCU has been studied extensively and led to reductions in the quantity and frequency of drinking by 50% throughout a 12-month follow-up period, when compared to a waitlist control group [57]. The DCU is 1 of the 2 web-based DHIs that have the strongest evidence supporting their efficacy based on RCTs [7].

In this study, the DCU was delivered via an ECA (namely eEVA) rather than via its traditional textual interface. As a BMI, the eEVA intervention combines MI style of communication with the common underlying elements of effective brief interventions characterized by the acronym FRAMES [2]: *Feedback* about client’s individual status is personalized and stored in a user model database, keeping a record of users’ answers for the next session(s); *Responsibility* for changing is left with the individual, and the language used throughout the intervention reflects this (eg, “I will not pressure you in any way”); *Advice* is provided in a supportive manner, with empathic choice of words and supportive body gestures (eg, leaning forward, head nodding); *Menus* of different options for changing that respect an individual’s readiness to change are offered; *Empathic* style of communication is central to the individual-clinician relationship, and it is conveyed by the ECA’s verbal utterances (eg, spoken reflections), nonverbal behavior (NVB; eg, smiling facial expressions, lean forwards, hand flips, nodding at appropriate times), and empathic choice of wording (eg, “It might be surprising to you to know that you are in the top percentile in drinking compared to people of your gender and age; you might want to review your answers again …”); and *Self-efficacy* is nurtured and emphasized throughout, including with choice of words and positive facial expressions (eg, various head nods and smiling facial expressions).

#### Technical Implementation of the Intervention

**Figure 3:**
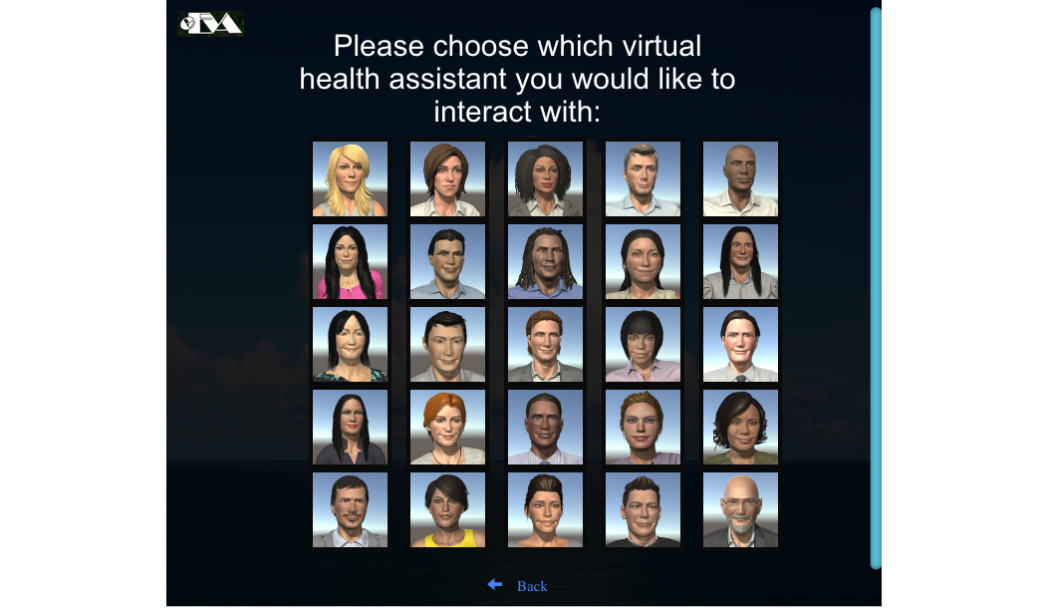
Menu of diverse empathic embodied virtual agent (eEVA) embodied conversational agents consumers can select.

Our eEVA framework (shown in Figure 2) provides (1) a realistic 3D WebGL graphics virtual environment with a realistic virtual office environment that can be “inhabited” by 1 of the 25 available racially diverse ECAs (shown in Figure 3), each with physiologically realistic Facial Action Coding System–validated facial expression animations and full body animations; (2) real-time speech recognition of the user’s answers; (3) text and multiple choice input; (4) voice synthesis for the ECA’s spoken utterances; (5) ECA’s lip synchronization between phonemes and visemes; (6) ECA’s adaptive nonverbal responses such as head nods or leaning forward depending upon the utterance dialog act; (7) configurable dialogue content; and (8) ability to capture and process users’ social cues such as facial expression recognition (which will be enabled in a future study).

Technically speaking, the framework consists of 3 main components. First, the application layer consists of a modular client-side JavaScript mainframe that controls the multimodal user interface, audio and video input, graphical user interface (GUI) interaction, and services such as speech recognition and speech synthesis. Second, the JavaScript mainframe handles execution of a scenario (the content of the DCU in this study) — a collection of state machines that are created by developers. Third, the scenario states can be constructed to pull information (eg, the ECA’s speech, graphics to show) from the data layer — a database of content.

The backbone of the client-side application is a JavaScript framework that handles the formation of a group of modules and the communication between them. Each module then implements various functionalities, including gaining feedback from the user (eg, asking to access microphone and camera) and processing input information (eg, analyzing users’ responses, extracting facial expressions if desired); determining how to answer the user (eg, words agent should say, NVBs); and answering the user through a multimodal 3D-embodied ECA, with speech synthesis, NVBs, and multimedia content (eg, text, images, and videos).

**Figure 4:**
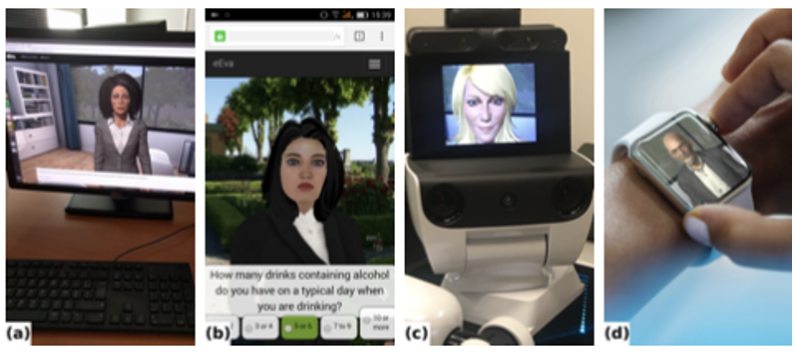
Empathic embodied virtual agent (eEVA) running on different platforms: (a) desktop, (b) mobile phone, (c) autonomous robot, (d) smartwatch concept.

This results in an interactive online application that can run on numerous platforms such as desktop, cell phone, autonomous robotic agent, and potentially smartwatch integrations (Figure 4). In addition, the user can also choose between a collection of 3D virtual characters to interact with — of different genders, races, and appearances. To personalize the eEVA system further, favorite chosen characters are remembered and displayed after login during the next interaction with the system. Distributing the framework core (eg, 3D character, perception, behavior) to consumer devices makes this technology scalable, with little to no overhead with additional users. Computer-intensive functionality such as speech and face recognition is asynchronous via web services or with built-in functionalities in the browser.

Unlike traditional ECAs, the eEVA design follows common modularity patterns found in robotics platforms such as ROS [60], allowing us to generate collections of modules to cover a diversity of application use cases, such as various browsers, many internet bandwidth limitations, and interaction capabilities. For instance, when using speech recognition, to provide the transcript of the user’s spoken utterances to be used by the ECA application, based on browser capabilities, a specialized module can be used to either interface with the Web Speech API or to use another service such as Watson Speech to Text. The advantage of this design is the seamless passing from one module implementation to another, including at runtime, without affecting the rest of the application.

To model social interaction, 3 main categories of module functionality are necessary, namely input/sensing modules (for perceiving social cues from the user in real time); social interaction decision-making modules*,* including an ECA behavior module, vocal command interpretation, and the scenario controller; and output/actuator modules for actually expressing verbal and nonverbal cues to the user. The functionalities of the main modules used in the current version of eEVA are listed in Table 1. Most modules have simple functions to retrieve or display information from and to the user or call functions from libraries (third-party or in-house) or services.

**Table 1:** Listing of the most significant modules and their function descriptions used in the empathic embodied virtual agent (eEVA) for our health agent.

Module	Function description
Input/Sensing Module	User microphone interface using WebRTC application programming interface (API) Speech recognition using Google Chrome API Interface with CoreNLP Graphic user interface (GUI) for direct user input (text, buttons)
Social Interaction Decision-Making Modules	Vocal command interpretation Embodied conversational agent’s (ECA) behavior (gesture and facial animations) Scenario controller (state machine execution)
Output/Actuator Modules	Speech synthesis 25 webGL 3D eEVA ethnically diverse characters

We tested 2 types of network connections: broadband and 4G mobile data. The majority of the launch time consists of loading the 3D character and surrounding virtual environment, which takes about 30 seconds and 25 seconds on 4G and broadband, respectively. The experiments (Table 2) showed that the main distributed functionalities of the eEVA framework allow real-time interaction and adequate loading times. This was echoed by users, as discussed in our Results section.

**Table 2:** Average response time and standard deviation analysis for the empathic embodied virtual agent (eEVA) using 4G or broadband connections over the internet between North America and Europe, with caching disabled (first run).

Functionality	Time on 4G mobile data (milliseconds)	Time on broadband internet (milliseconds)
Unity 3D character, mean (SD)	30018 (663)	24626 (1910)
TTS^a^ (sentence), mean (SD)	939 (381)	551 (141)
TTS (word), mean (SD)	72 (40)	44 (23)
Speech recognition	~30 (offline processing)	N/A^b^
Entire HTTP request, mean (SD)	1124 (166)	784 (66)
DOM^c^ loading, mean (SD)	2313 (80)	1635 (224)

^a^TTS: text to speech.

^b^N/A: not applicable.

^c^DOM: Document Object Model.

#### Model of Empathic Verbal and Nonverbal Behavior

To simulate some of the communication psycholinguistic signals of a counselor delivering a BMI, we first videotaped BMI sessions between a live licensed counselor and a client. Then, a clinical expert reviewed the videotapes to code verbal reflections and NVB. From these, the expert generated a set of rules for basal behaviors of the health agents. Based on the codes of verbal and NVBs, eEVA was implemented with the following verbal reflections: “Ah.” “Alright.” “Okay.” “Good.” “Sounds Good.” “Oh, okay.” “Great!” “Thanks for letting me know.” “Oh, I see.” “Okay, thanks!”

In addition, the following NVB animations were synthesized on the agent’s face: smile, facial expressions, hand gestures (typing on a computer at a desk, hands resting on the agents’ legs, formless flick, two-handed flip, two-handed contrast gesture), body leans (forward), head gestures (nod, shake, nonshake), and eyebrow movements (up, neutral, and down), which our results (discussed later) showed are conducive of a positive experience for the user with the agent in the given health care context.

Since it was determined that head nods are critical to portraying (some level of) active listening, we sought to offer 3 variants depending on the user’s chosen responses. We created a set of nods using established emotional expressions governed by activation of specific individual facial movement animations. All 3 of the head nods included activation of head down and eyes down. However, depending on the type of reaction required, these also included facial expressions (eg, smile, mild concern).

The patterns observed in the videotapes of the counselor-client session also directed us towards creating rules about when certain statements should be made, to ensure the counselor did not appear judgmental and to make the interaction appear more natural. In all scenarios, the counselor began seated at the desk while typing on the computer, then looked up and moved to the chair closer to where the user perceives they are sitting during an initial greeting. Once seated, the counselor began with a greeting introduction and then moved on to delivering the DCU. Verbal responses to user responses were applied based on the “score” of each question to provide nonjudgmental reactions for higher scores that might indicate a problem and positive reactions for scores that might suggest healthy consumption levels. For example, “Sounds good” was used in response to a user mentioning that they wanted to change.

**Figure 5:**
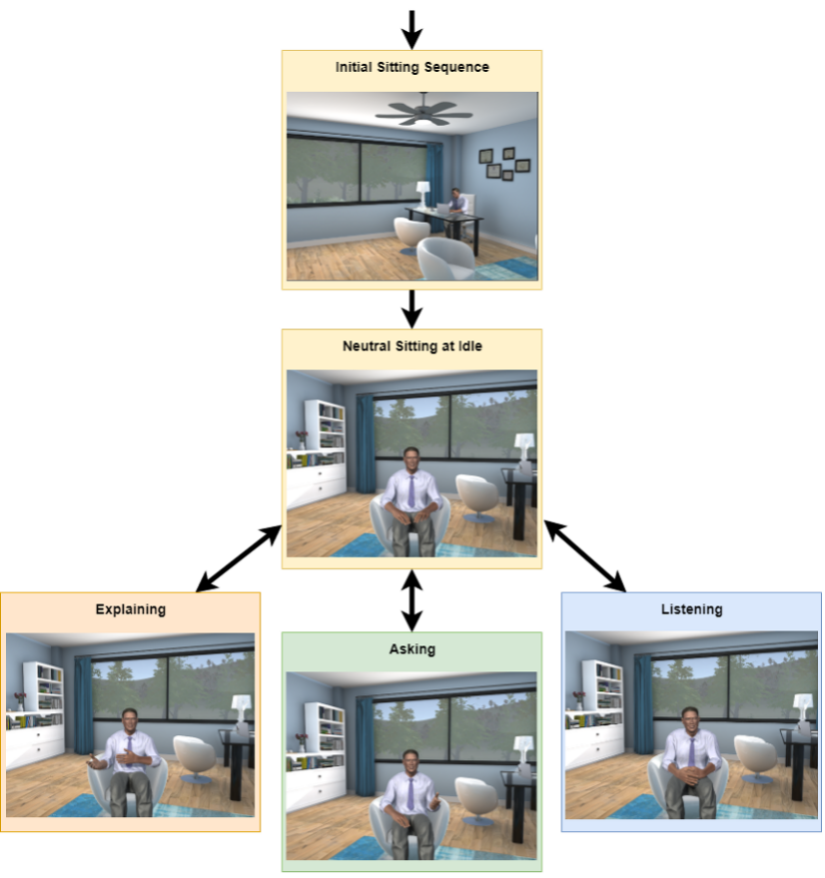
Defining nonverbal behaviors for virtual health agents. All undergo the initial sitting sequences and then assume a neural sitting at idle position. From here, the agent can enter either the be explaining, asking, or listening loop.

In parallel, NVBs were applied using a set of states that were determined as appropriate given the context of the interaction as shown in Figure 5: Neutral, Explaining, Asking, and Listening. In the *Neutral* state, the counselor spoke and used a smile, a gaze, facial expressions, head gestures, or eyebrow movements. In the *Explaining* state, which was activated during long periods of speaking, the two-handed flip and two-handed contrast gesture were applied. When the counselor posited a question to the user, the *Asking* state was initiated, which included a single hand formless flick. While the counselor waited for the user to respond to a question, the *Listening* mode was initiated, which included a leaning forward gesture. The leaning gesture remained in effect until a choice was made, at which point the body resumed an upright sitting position.

### Participants

Participants were alcohol users aged 21 to 55 years and recruited online to participate in the intervention. Participants had to have engaged in heavy drinking (consumed 5 drinks in one sitting at least once in the past year), not currently be receiving treatment for their AUD, and not have a medical condition for which alcohol use would be contraindicated. Users were also screened for severe mental illness. We recruited 51 participants as part of a larger RCT of the effectiveness of this program in reducing alcohol abuse. Participants were 62% (32/51) male, were 32% (19/51) female, and had a mean age of 28 (SD 15.8) years. Participants reported their race as White (21/51, 42%), Black (12/51, 24%), Asian (3/51, 7%), Other (2/51, 3%), and no response (12/51, 24%). Participants reported their ethnicity as non-Hispanic (43/51, 84%), Hispanic (8/51, 15%), and no response (2/51, 1%). Marital status was reported as married (23/51, 46%), single (17/51, 34%), divorced (3/51, 7%), widowed (1/51, 1%), or no response (6/51, 12%). Their education level was reported as high school (7/51, 14%), some college (13/51, 25%), Associate’s degree (10/51, 19%), Bachelor’s degree (18/51, 36%), and Graduate degree (3/51, 6%).

### Procedures

Participants were recruited online via targeted advertising on Facebook, offering free treatment for alcohol users and compensation for research participation. When users clicked on the ad, they were redirected to an online screener (on Qualtrics) to ensure they were eligible for the study. If they were eligible, users were randomly assigned to receive the same DCU MI intervention delivered online either by an ECA (eEVA) or a textual interface. Participants in this study were those who were assigned to eEVA, since the control group assigned to the text-only interface could not comment on the ECA’s social features that they did not see. Once randomized, participants were provided with a username and password to enter the DHI platform eEVA. Participants had to log on, enter demographic information, and begin the intervention. After completing the intervention (participants were given 1 week to complete), they were redirected to an online survey on Qualtrics to provide feedback about their experience with the intervention.

### Measures

After completing the intervention, participants were asked to provide feedback on the feasibility, acceptability, and utility of the technology. Specifically, we sought feedback regarding their engagement (acceptability), perceived utility, and intent to use the technology (feasibility). Participants completed a questionnaire developed for this study. Since there does not exist, to date, a standardized instrument to evaluate interaction with ECAs of various levels of complexities, we used and adapted relevant existing questionnaires commonly used for the evaluation of human interaction with technologies involving some social cues, whether embodied with graphics or with robot technologies. Questions were based on a combination of the engagement model by O’Brien and Toms [61], Almere model by Heerink et al [62], and “Godspeed questionnaire” by Bartneck et al [63], which has been widely used to evaluate human-technology interactions using 5 key concepts — anthropomorphism, animacy, likability, perceived intelligence, and safety — that have been found useful for interacting with either ECAs or robots. All responses were on a 7-point Likert scale (1=Strongly Agree; 7=Strongly Disagree), with lower scores indicating more desirable findings. There was no cut-off as the measures were combined for the purpose of this study and were meant to provide descriptive feedback. In addition, each question included a blank space with a prompt of “Comments” for participants to provide optional qualitative feedback to each question.

The engagement model by O’Brien and Toms [61] explores acceptability of the ECA via constructs of user engagement with technology. Six attributes of a technology make it more likely that a user will engage with it (challenge using the technology, interest, motivation to use the technology, and appeal of the technology [eg, “I found the health assistant interesting”]). Responses were on a 7-point Likert scale (1=Strongly agree; 7=Strongly disagree).

The Almere model evaluates the user’s acceptance of the digital health agent by relying on constructs from the Unified Theory of Acceptance and Use of Technology [12]. Users replied to 13 statements (eg, “I enjoyed participating in this session with the health assistant”) that map to constructs that predict intent to use the technology and perceived usefulness of the technology. Responses were on a 7-point Likert scale (1=Strongly agree; 7=Strongly disagree).

The Godspeed questionnaire consists of 12 questions that capture 5 constructs that measure human-like traits of robots, which we adapted for ECA (eg, “The health agent seemed warm”): (1) anthropomorphism (eg, moving rigidly or moving elegantly), (2) animacy (eg, mechanical or organic), (3) likeability (eg, unfriendly or friendly), (4) perceived intelligence (eg, incompetent or competent), and (5) perceived safety (eg, anxious or calm). Each trait could be rated as being very human-like to very unhuman-like on a 7-point scale.

All questions included a fill-in option for participants to expand on their numerical responses with qualitative feedback if they wanted to. We highlight some of those comments in the Results section.

## Results

### Acceptability and Utility

Participants reported high acceptability and utility of the technology, as indicated by their scores on the Almere model questions (mean 2.31, SD 1.05). Most participants reported enjoying their interaction with the agent (44/51, 86%) stating “The questions she asked me, no one had asked me before and helped recognize my drinking problem.” They thought the agent was both physically appealing (38/51, 74%) and had a pleasant voice (42/51, 82%), stating:

He is neat, he has a good haircut, he is well dressed.

He appears wise, intelligent, and healthy.

The voice was adequate, calm, and confident.

Furthermore, participants indicated that they found it easy to interact with the health agent (45/51, 89%) and they learned to do so quickly (45/51, 89%), stating:

I just followed the instructions and voila!

I just waited and followed his instructions; it was easy.

Participants had more difficulty with the voice feature of the technology, with only 69% (35/51) reporting that they felt like the agent understood them when they spoke into the microphone. Based on the qualitative feedback, this may have been due to issues with participants’ microphone setting rather than the technology itself:

I set my microphone up, but it seems there is a problem with it.

I tried to speak my answers, but it never worked so I ended up typing them.

Those who were able to get their microphones to work seemed to have no difficulty speaking to the agent as echoed by their qualitative feedback (eg, “I don’t even repeat my answers; the agent understands me very well.”). Despite some difficulties with the microphone, 78% (40/51) felt like they could have a conversation with the agent, stating “I felt that he knows me, knows what I want” and reported that they sometimes felt like they were talking to a real person (36/51, 71%), stating that:

…his voice sounded quite real…

…the gestures he made, way he moved around…

he answered me like a real person…

Participants further reported that the health assistant was friendly (45/51, 89%) and they found it to be useful (44/51, 88%) because:

…he explained things that I did not know…

…it helped me recognize that I have a problem…

I learned a lot.

Participants reported that they were comfortable disclosing information about their drinking to their digital health assistant (48/51, 93%), with 83% (41/51) reporting that they were *more* comfortable disclosing their drinking to the digital health assistant over their medical doctor. Indeed, participants highlighted:

The assistant gives me a level of trust that I don’t have with other humans.

It is more easy talking to (the health agent) than to a real person.

I don’t feel like they are judging me.

Finally, participants reported trusting the advice the health agent gave them (43/51, 85%) and that they planned on following that advice (44/51, 86%), stating:

I think she is sincere and wants to help me with my problems.

…because it is based on facts and studies and that is real and valid information for me.

One participant noted “I didn’t feel like I was given advice, more like information to be able to make my own decision. I was the one with the power to give myself advice.” *—* perfectly capturing the intent of MI.

### Engagement

Participants were highly engaged with the DHI, as indicated by their score on the engagement questions (mean 2.86, SD –0.96), indicating that the majority of participants agreed with statements around how engaged they were. Specifically, 69% (35/51) were not worried about making mistakes while using the technology, stating:

At first, it was a little bit intimidating, but then I felt confident.

The assistant feels understanding, attentive, very friendly.

A majority (44/51, 86%) thought it was a good idea to use the health assistant, reporting:

He is kind of like a home counselor who works with reliable information and statistics.

It is practical, easy to use, and guides the person on what to do without forcing us to make a final decision.

Participants felt that the system could be adaptive to their needs (46/51, 90%), stating that “it could be adapted to other health problems like smoking*.*”

Finally, 88% (45/51) found the health assistant to be interesting, indicating “I was impressed by the way it converts my answers into figures and important information for my health” and said they would interact with the agent again (43/51, 85%):

Setting a new exchange with the health assistant would help me to reach my goal.

### Impressions of the Digital Health Agent

Participants reported a high number of human-like traits on the Godspeed questions (mean 2.07, SD 0.89). Participants reported that the agent moved appropriately (43/51, 85%) and seemed warm (46/51, 90%), responsive (45/51, 89%), knowledgeable (47/51, 92%), relaxed (46/51, 90%), flexible (42/51, 83%), honest (46/51, 90%), respectful (46/51, 90%), confident (47/51, 92%), interested (44/51, 86%), open-minded or nonjudgmental (43/51, 84%), and supportive (45/51, 89%).

Overall, participants’ responses to the questionnaires and qualitative feedback indicated that they found the delivery of an MI intervention by a digital health agent over the internet to be acceptable, be engaging, and have features that are close to human-like.

## Discussion

Our goal with this study was to understand if the technology we developed was feasible (able to be implemented online), acceptable, useful, and easy to use by consumers. As such, we focused largely on the technology aspects of the intervention.

### Principal Findings

This study provides an optimistic outlook for the use of digital health agents to deliver brief online interventions in the future. Consumers overwhelmingly reported positive experiences in their interactions with the agent, with many reporting that they trusted the agent and felt that they could more comfortably disclose information that they may not have disclosed to a human provider. This echoes what has been found in the literature around disclosing to computers versus humans [12,14,64,65].

Further, participants tended to attribute many human-like traits to their agent (eg, friendly, trustworthy, kind) and commented positively on the physical appearance, voice, and physical gestures of the agent. Our team engaged in coding of verbal reflections, hand gestures, and facial expressions of a real therapist to enhance the digital agent’s nonverbal communication to resemble what a therapist might do in session [66].

Implications for this work are important. Given the shortage of mental health workforces in many locations, digital health agents may provide an acceptable complement to traditional face-to-face therapy, reducing demand for higher levels of care, where a digital health agent can act as a clinician “extender” to deliver booster sessions. Similar to telehealth services, digital health agents resolve a number of barriers to care such as transportation and scheduling.

In addition, digital health agents reduce stigma around mental health care, are less costly than one-on-one therapy, and can be scaled out and disseminated. For individuals with high levels of social anxiety, digital health agents may provide them with a unique opportunity to get help. Given the digital nature of the agent, it is possible to adjust the programming to make the agent able to speak in multiple languages, reducing language barriers for minority and refugee populations. Already, consumers can pick a digital health agent from a library of diverse options of physical visual features (gender, age, race, and ethnicity; see Figure 2) and vocal features (gender). This is an exciting development given the lack of a diverse mental health workforce [67,68].

This study has provided the investigative team with valuable feedback to improve the technology, including improving the flow and tone of the voice, providing questions in text, and formatting the technology for use on mobile phones and with lower bandwidth.

### Limitations

Despite these enthusiastic findings and implications, it is important to note a number of limitations. First, digital health agents cannot replace traditional therapy and certainly cannot manage crisis situations. They are well suited for brief, structured interventions, but cannot replace the complex nature of a therapeutic relationship and complex therapeutic interventions such as family therapy and emotion-focused therapy. They were considered and studied in this article as clinician “extenders.” Second, this study was conducted with participants in the United States only. It is unclear if technology acceptability would be as high in other countries. Third, the impact of the DHI on actual alcohol outcomes remains unclear. A study is underway to better understand the effects of a digital health agent. Fourth, it is unclear whether ECAs are suitable for various health problems besides alcohol and for various other populations not studied here, such as the elderly or children. Finally, the access to and cost of reliable internet necessary to use ECAs may limit access to some — potentially further increasing the digital divide.

### Conclusions

This type of intervention and research on digital health agents in virtual reality over the internet are still in their infancy, and there is much work to be done. For instance, the same BMI intervention delivered in this study could be adjusted to other health behaviors (eg, other substances, medication compliance, weight management). Other interventions could be delivered to screen or treat a variety of problems. Furthermore, our team is working on integrating natural language dialog management features such that the agent will respond to the consumer’s answers without needing specific input from which to read. We conducted preliminary work [26] for a speech-enabled ECA for BMI interventions with promising results. However, natural language understanding is still a very open research area of computer science, and its use in DHIs is not robust enough to deploy with real users who need support and help, without the potential frustrations generated by unreliable agent’s speech understanding. Our team has also worked on features based on AI that allow a PC-based agent (ie, operating on PC only and not web-based) to pick up on the consumer’s facial expressions in real time (eg, if the consumer smiles, the agent smiles back), which has been shown to improve users’ engagement with digital health agents [25], and we plan to add this type of feature to our web-based eEVA system.

Despite all these exciting potential developments, it is critical to evaluate DHIs with high levels of rigor before they can be deployed for the population at large. As pointed out by Carroll [7], very few of the many available internet-based interventions have been carefully evaluated in well-controlled clinical trials, and the majority of those studies have been conducted with college populations, bringing into questions the generalization of the results to broader society. The conclusions that can be drawn from many studies are constrained by high levels of dropout, high attrition, and weak control conditions (eg, waitlists). To that end, we will report on the results of the RCT we conducted to assess the efficacy of the eEVA DHI compared to a text-only version of the intervention.

We furthermore consider that these digital health agents and DHIs can only complement the unique experience of psychosocial therapy and serve as “clinician extenders” [7]. As suggested by Mohr et al [21], mental health technologies in general should be considered as sociotechnical systems (or technology-enabled services rather than mere products) that must fit within an ecosystem of mental health services (involving human support and organizational factors). Our aim is to provide relief to a clogged mental health system and provide online access to self-help to individuals who otherwise would not access traditional face-to-face care. Further research on whether DHIs increase access to care by removing the barriers identified earlier (eg, availability and proximity of trained providers, affordability, stigma) or whether they increase the digital divide is needed [21]. Digital health agents, even with integrated AI, will not replace human therapists; they should be considered therapist extenders.

## Abbreviations

AI: artificial intelligence
AUD: alcohol use disorder
BMI: brief motivational interviewing
DCU: Drinker’s Check-Up
DHI: digital health intervention
ECA: embodied conversational agent
eEVA: empathic embodied virtual agent
GUI: graphical user interface
MI: motivational interviewing
NVB: nonverbal behavior
RCT: randomized controlled trial
TAM: technology acceptance model
